# Plant breeding involving genetic engineering does not result in unacceptable unintended effects in rice relative to conventional cross‐breeding

**DOI:** 10.1111/tpj.14895

**Published:** 2020-07-19

**Authors:** Qingsong Liu, Xiaowei Yang, Vered Tzin, Yufa Peng, Jörg Romeis, Yunhe Li

**Affiliations:** ^1^ State Key Laboratory for Biology of Plant Diseases and Insect Pests Institute of Plant Protection Chinese Academy of Agricultural Sciences Beijing 100193 People’s Republic of China; ^2^ College of Life Sciences Xinyang Normal University Xinyang 464000 People’s Republic of China; ^3^ French Associates Institute for Agriculture and Biotechnology of Drylands Jacob Blaustein Institutes for Desert Research Ben‐Gurion University of the Negev Sede Boqer Campus Midreseht Ben Gurion 8499000 Israel; ^4^ Agroscope, Research Division Agroecology and Environment Zurich 8046 Switzerland

**Keywords:** *Oryza sativa*, genetic engineering, unintended effect, transcriptome, metabolome

## Abstract

Advancements in ‐omics techniques provide powerful tools to assess the potential effects in composition of a plant at the RNA, protein and metabolite levels. These technologies can thus be deployed to assess whether genetic engineering (GE) causes changes in plants that go beyond the changes introduced by conventional plant breeding. Here, we compare the extent of transcriptome and metabolome modification occurring in leaves of four GE rice lines expressing *Bacillus thuringiensis* genes developed by GE and seven rice lines developed by conventional cross‐breeding. The results showed that both types of crop breeding methods can bring changes at transcriptomic and metabolic levels, but the differences were comparable between the two methods, and were less than those between conventional non‐GE lines were. Metabolome profiling analysis found several new metabolites in GE rice lines when compared with the closest non‐GE parental lines, but these compounds were also found in several of the conventionally bred rice lines. Functional analyses suggest that the differentially expressed genes and metabolites caused by both GE and conventional cross‐breeding do not involve detrimental metabolic pathways. The study successfully employed RNA‐sequencing and high‐performance liquid chromatography mass spectrometry technology to assess the unintended changes in new rice varieties, and the results suggest that GE does not cause unintended effects that go beyond conventional cross‐breeding in rice.

## Significance Statement

We compared the biological variation at mRNA and metabolite levels among four genetically engineered rice lines and nine conventionally bred rice cultivars that represent a range of genetic and phenotypic diversity in rice. The results provide evidence that genetic engineering does not cause unintended effects that go beyond conventional cross‐breeding in rice.

## INTRODUCTION

Genetic engineering (GE) is widely used to introduce desirable traits such as insect resistance, disease resistance, herbicide tolerance, drought tolerance and improved nutrition in crops (ISAAA, 2018). Amongst, the insect‐resistant GE (IRGE) crops producing insecticidal proteins, crystal and vegetative insecticidal proteins derived from the bacterium *Bacillus thuringiensis* (Bt) have been widely grown worldwide (ISAAA, 2018). During 2018, in total, 104 million hectares of Bt‐transgenic IRGE crops including soybean (*Glycine max*), cotton (*Gossypium* species), maize (*Zea mays*) and, to a small extent, eggplant (*Solanum melongena*) and sugarcane (*Saccharum officinarum*) were planted (ISAAA, 2018). The wide growth of Bt crops has provided area‐wide suppression of some major lepidopteran crop pests such as pink bollworm (*Pectinophora gossypiella*) (Carrière *et al*., 2003), cotton bollworm (*Helicoverpa armigera*) (Wu *et al*., 2008), European corn borer (*Ostrinia nubilalis*) (Hutchison *et al*., 2010; Dively *et al*., 2018) and corn earworm (*Helicoverpa zea*) (Dively *et al*., 2018), leading to a significant decrease of broad‐spectrum chemical insecticide application (Klümper and Qaim, 2014; NASEM, 2016; Brookes and Barfoot, 2018; Li *et al*., 2020).

In contrast to the general acceptance of crops obtained by conventional breeding and associated food products, IRGE crops are subjected to rigorous evaluation. Common concerns are the potential adverse effects on the environment and on human health. The latter, in particular, appears to be an important factor delaying the commercial use of IRGE crops in many countries, including China (Li *et al*., 2020). Sources of potential harm caused by GE plants can be separated into two broad categories of change, i.e., intended changes and unintended changes (Ladics *et al*., 2015). Both are addressed in the pre‐market risk assessment for any novel GE crop. As the intended changes in a GE plant concern the introduced genetic material and the related desired trait, in general, the associated risks can be anticipated and assessed (Ladics *et al*., 2015). In contrast, unintended changes can hardly be anticipated and are difficult to be detected raising caution when assessing the risk of GE plants (Ladics *et al*., 2015; Schnell *et al*., 2015; NASEM, 2016; Wang *et al*., 2018b). Unintended changes could be materialized because of gene insertion, random mutation, somaclonal variation, pleiotropy, position effect, or the tissue culture process during the development of GE plants (Miki *et al*., 2009; Ladics *et al*., 2015; Schnell *et al*., 2015). Typically, unintended changes are addressed by profiling the GE plant using compositional analysis (Herman and Price, 2013) and phenotypic characterization (Horak *et al*., 2007).

The advancements in omics‐based systems biology including genomics, transcriptomics, proteomics and metabolomics profiling have greatly enhanced the possibilities to analyze unintended changes in plants and these techniques have been shown to be powerful approaches for identifying changes in GE plants, as has been demonstrated for example for Arabidopsis, rice, maize, soybean, barley and pigeon pea (Kuiper *et al*., 2001; Ouakfaoui and Miki, 2005; Ricroch *et al*., 2011; Gong and Wang, 2013; Herman and Price, 2013; Wang *et al*., 2018b; Tan *et al*., 2019). However, in most study cases comparative analyses were restricted to one GE line and its closest non‐GE counterpart. These results commonly revealed certain differences in the transcriptomes, proteomes and metabolomes of the tested plants (Ricroch *et al*., 2011; Gong and Wang, 2013; Wang *et al*., 2018b). However, such results can hardly tell whether such differences are specific to plant GE breeding other than conventional breeding and whether they represent safety problems and their value for risk assessment is thus questionable (Raybould and Macdonald, 2018). So far, little effort has been made to compare the potential unintended effects brought by GE and by conventional plant breeding approaches (Batista *et al*., 2008; Gong *et al*., 2012; Wang *et al*., 2018b). Recently, a tiered evaluation strategy for analyzing unintended changes in crops using ‐omics technologies was proposed by experts from the National Academics of Science, Engineering and Medicine, which highly recommend that unintended changes in a new GE variety should be evaluated by comparison with a set of conventionally bred cultivars, but not just with their parental isolines (NASEM, 2016).

Rice (*Oryza sativa*) is an important staple food for more than half of the population in China (Li *et al*., 2020). Rice production is constrained by a complex of insect pests, among which lepidopterans such as the rice striped stem borer (*Chilo suppressalis*), yellow stem borer (*Scirpophaga incertulas*) and rice leaf roller (*Cnaphalocrocis medinalis*) can cause substantial yield losses (Li *et al*., 2020). To control these pests in an efficient and environmentally friendly way, dozens of IRGE rice lines expressing Bt proteins, have been developed in China, and multiple lines exhibited high efficacy in target pest control (Chen *et al*., 2006, 2011; Liu *et al*., 2016; Li *et al*., 2020). When assessing the risks of Bt rice, most studies were target‐oriented and focused on the intended effects, while little attention was paid to the unintended effects (Xue *et al*., 2012; Li *et al*., 2017; Fu *et al*., 2019).

**Figure 1:**
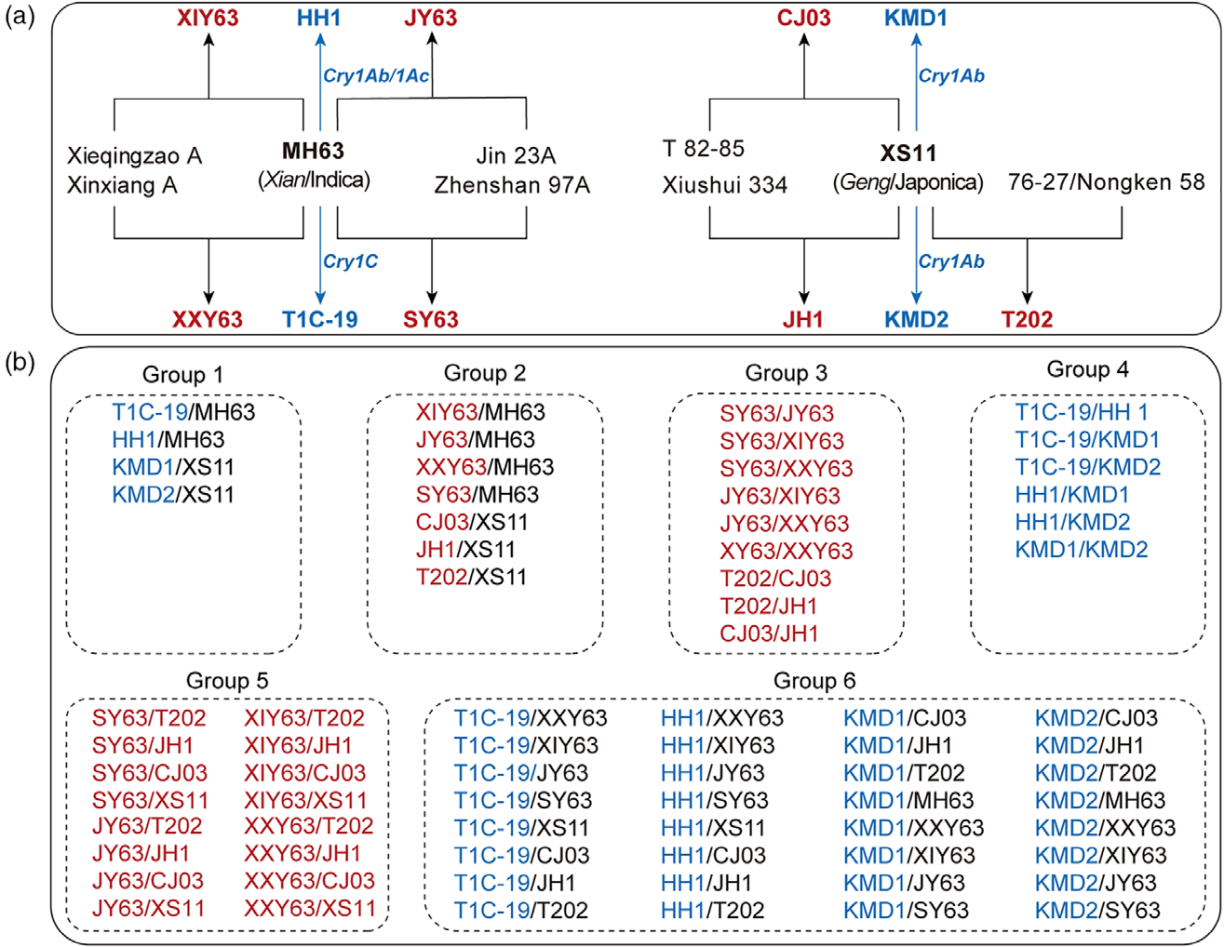
Genetic relations among the studied rice lines and grouping comparison design for the analyses. (a) Genetic relations among the studied rice lines. (b) Experimental design for pairwise comparisons for gene expression and metabolite accumulations between different rice lines. Group 1, comparisons between Bt rice lines and their non‐Bt parental rice plants; group 2, comparisons between conventional cross‐breeding rice lines and their parents; group 3, comparisons between conventional cross‐breeding rice lines with the same parents; group 4, comparisons between Bt rice lines; group 5, comparisons between conventional cross‐breeding rice lines; group 6, comparisons between Bt rice lines and conventional cross‐breeding non‐Bt rice lines. HH1: Huahui No.1, MH63: Minghui 63, KMD1: Kemingdao 1, KMD2: Kemingdao 2, XS11: Xiushui11, XY63: Xieyou 63, JY63: Jinyou 63, XXY63: Xinxiangyou 63, SY63: Shanyou 63, CJ03: Chunjiang 03 jing, JH1: Jiahua 1, T202: Tai 202. Rice lines with red, blue and black colour represent the conventional cross‐breeding lines, GE lines and the parents of conventional cross‐breeding lines or GE lines, respectively. Bt, *Bacillus thuringiensis*; GE, genetic engineering.

In the current study, we compared the biological variation at mRNA and metabolite levels among four GE rice lines and nine conventionally bred rice cultivars that have a close genetic relation with the GE rice lines (Figure 1). We performed two of the omics‐based systems biology approaches including transcriptomics using RNA‐sequencing (RNA‐seq) and metabolomics using high‐performance liquid chromatography mass spectrometry (HPLC‐MS), and investigated these datasets. Based on these results, the potential unintended effects caused by two different plant breeding methods were analyzed comparably.

## RESULTS

### Evaluating the rice lines transcriptome

We decided to analyze leaf material, as leaves are important plant organs due to their role in many important biological functions such as photosynthesis, respiration and transpiration. In total, 39 RNA‐seq libraries were constructed, resulting in approximately 22–51 million clean reads per library; the guanine‐cytosine content accounted for 52%–57% of these reads (Table S1). Using the rice IRGSP‐1.0 as a reference genome, 90%–96% of the clean reads were mapped, with the unique mapping rates ranging from 88% to 94%. Gene structure analyses showed that most of the mapped reads (91%–94%) were distributed in exons (Table S1). These results suggested that the transcriptome datasets, generated from 13 rice lines, is sufficient for further analyses of comparison within and between the different lines.

### Analyzing gene expression

**Figure 2:**
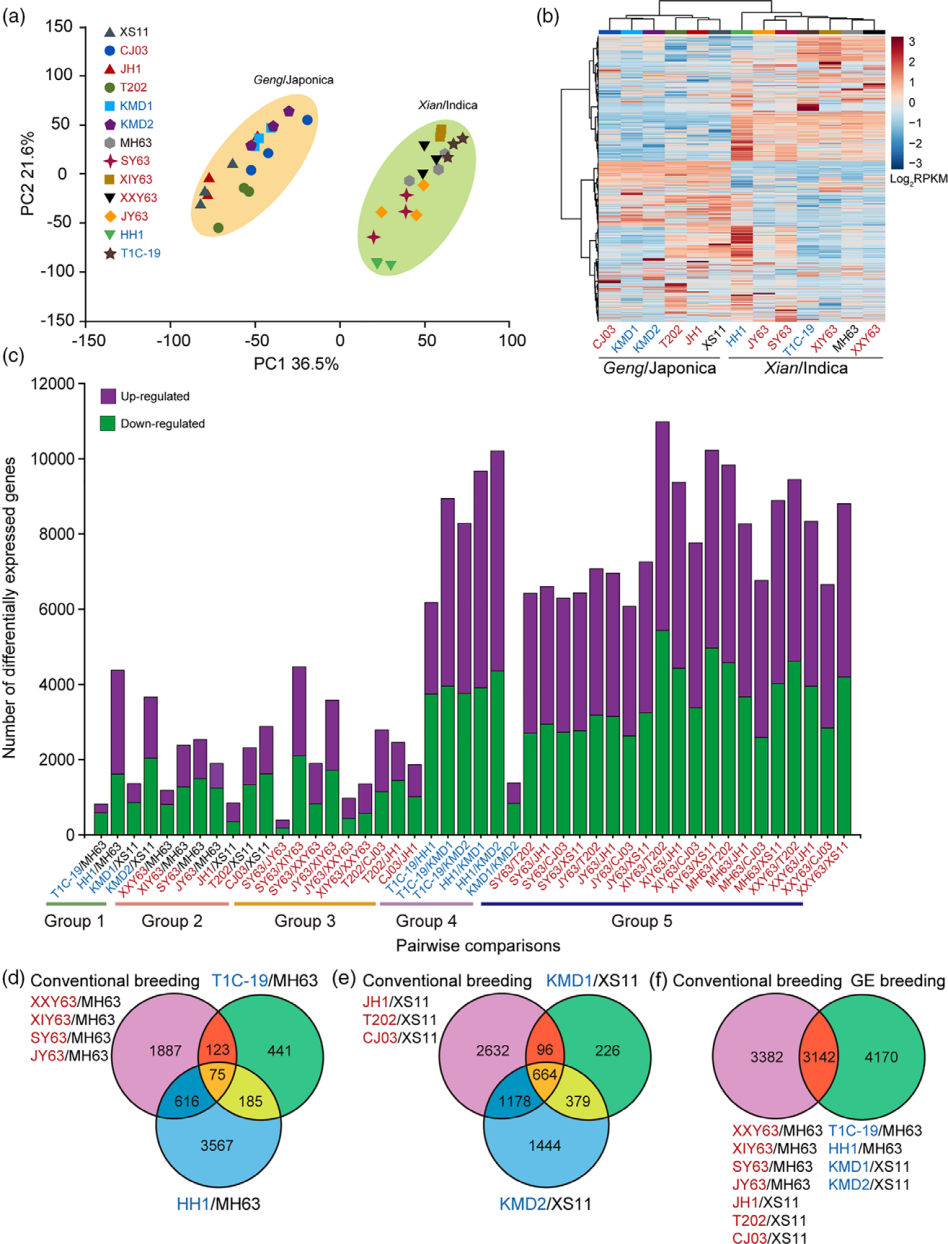
Overall description of transcriptome data. (a) Principal components (PCs) analyses of gene expression levels in leaves of 13 rice lines. Score plot of the first two PCs with the explained variance. (b) Hierarchical clustering of 13 rice lines using the total detected gene expression data. In the heatmap, each rice line is visualized in a single column and each gene is represented by a single row. Gene expressions are shown in different colors, where red indicates high abundance and low relative expression is shown in blue (color key scale right of the heatmap). (c) Pairwise comparisons of differentially expressed genes (DEGs) between different rice lines. (d–f) Venn diagrams depicting the unique and shared DEGs among *Xian*/Indica subspecies (d), among *Geng*/Japonica subspecies (e), and between lines obtained by conventional breeding or genetic engineering (GE) breeding (f). RPKM, reads per kilobase of transcript, per million mapped reads.

The RNA‐seq dataset was normalized to fragments per kilobase of transcript per million mapped read values to quantify the levels of gene expression, including 44 425 genes (Table S2). A principal components analysis (PCA) was performed on all 39 transcriptomic datasets to obtain a global view of the gene expression across the 13 rice lines. As shown in Figure 2(a), the first two principal components (PCs) explain 36.5% (PC1), and 21.6% (PC2) of the total variance, respectively. PC1 revealed a clear separation between *Geng/*Japonica (GJ) rice lines (Xiushui 11, XS11; Chungjiang 03 jing, CJ03; Jiahua 1, JH1; Tai202, T202; Kemingdao1, KMD1; Kemingdao 2, KMD2) and *Xian/*Indica (XI) rice lines (Minghui63, MH63; Shanyou 63, SY63; Xieyou 63, XIY63; Xinxiangyou 63, XXY63; Jinyou 63, JY63; Huahui no. 1, HH1; T1C‐19). However, for both rice subspecies (GJ and XI), the first two PCs could not separate the GE lines or conventionally bred rice lines from their parental lines, and GE lines from the conventionally bred lines. Consistently, rice lines belonging to the GJ or XI subspecies were clustered in the same class in a hierarchical way, respectively (Figure 2b).

Subsequently, differentially expressed genes (DEGs) of the 13 rice lines based on the different grouping comparisons described in Figure 1 were screened, showing distinct differences in gene expression among the lines. In total, 394–10 980 DEGs ranging from 1.29% to 33.60% were detected in the total genes among the 78 group comparisons (Figure 2c, Figure S1 and Table S3). Pairwise comparisons showed that the percentage of DEGs on the total detected genes (%) ranged from 2.63 to 14.57 between a Bt line and its non‐Bt parental line, from 2.81 to 9.31 between a conventional breeding line and its parental line, from 1.29 to 14.49 between each of the conventionally bred lines with the same parental line, from 4.39 to 31.73 between Bt rice lines, and from 18.53 to 33.60 between all non‐GE rice lines. Specifically, the number of DEGs between T1C‐19 and MH63 were 824, of which, 590 were upregulated, and 234 were downregulated. More DEGs were downregulated than upregulated in the comparison between HH1 and MH63, while more genes were upregulated than downregulated in the group comparisons of KMD1/XS11 and KMD2/XS11. Similarly, a higher proportion of upregulated DEGs were also found in conventional cross‐breeding rice lines compared with their parents except for the comparison between JH1 and XS11, of which 356 genes were upregulated and 492 genes were downregulated. The number of DEGs between GE lines and non‐GE parental rice lines were, in most cases, less than those between GE rice lines and conventionally cross‐breeding rice lines (Figure S1a). Overall, the number of DEGs between Bt rice lines and their parents were within the normal range of gene expression changes among non‐GE rice lines (Figure 2c).

The distribution of DEGs was calculated for each comparison and presented in Venn diagrams (Figure 2d–f). The DEGs in the grouped comparison between conventional breeding rice lines and their parent were pooled together as one group because these lines were all developed by conventional cross‐breeding and have been commercialized in China for many years. As shown in Figure 2(d–f), the distribution of DEGs was genotype‐specific. Although a large number of DEGs was detected in pairwise comparisons, when compared with their common parents, there were still a number of genes (75 for XI rice lines and 664 for GJ rice lines) expressed that were consistently different among lines developed by conventional cross‐breeding and GE breeding (Figure 2d,e). We also compared the distribution of DEGs between rice lines developed by conventional cross‐breeding and GE breeding regardless of their genetic background, and found that the two breeding methods shared 3142 DEGs (Figure 2f). These results suggest that both conventional cross‐breeding and GE breeding methods could change the expression of non‐target genes.

### Functional enrichment analysis of DEGs

To gain more insights into the function of DEGs among different comparisons, we conducted Gene Ontology (GO) and Kyoto Encyclopedia of Genes and Genomes (KEGG) pathway enrichment analyses of the DEGs in a total of 44 pairwise group comparisons. Interestingly, no significantly enriched biological process GO terms was found in 15 pairwise group comparisons (Table S4). In the remaining 29 comparisons, different biological process terms were enriched in specific comparisons, with DNA integration as the most popular GO terms (Table S4). Similarly, KEGG enrichment analyses indicated that no significantly enriched pathway term was found in 26 pairwise group comparisons (Table 1). In the remaining 18 comparisons, the DEGs are involved in different pathways, with ribosome as the most popular pathway term (Table 1). Specifically, in group 1, there were no significant enriched pathways of the DEGs in the comparisons of T1C‐19/MH63, HH1/MH63 and KMD2/XS11. Photosynthesis was the only significant enriched pathway of DEGs between KMD1 and XS11 (Table 1). In group 2, the DEGs mainly involved in diterpenoid biosynthesis, phenylalanine metabolism, phenylpropanoid biosynthesis and mitogen‐activated protein kinase signaling pathway‐plant. In group 3, there were no significantly enriched KEGG terms of DEGs in most comparisons, with plant–pathogen interaction and phenylalanine metabolism, phenylpropanoid biosynthesis and phenylalanine metabolism, and plant–pathogen interaction are significantly enriched in the comparisons of XIY63/XXY63, T202/JH1 and CJ03/JH1, respectively. In groups 4 and 5, there were no significantly enriched KEGG terms of DEGs in most comparisons.

**Table 1:** Kyoto Encyclopedia of Gene and Genomes (KEGG) pathway enrichment analyses of differentially expressed genes (DEGs) of pairwise comparisons between different rice lines

Group	Comparisons	KEGG ID	Description	Adjusted *P* value	Number of DEGs
Group 1	T1C‐19/MH63	–			
	HH1/MH63	–			
	KMD1/XS11	osa00195	Photosynthesis	1.37E‐03	7
	KMD2/XS11	–			
Group 2	XXY63/MH63	osa00904	Diterpenoid biosynthesis	1.16E‐02	4
	XIY63/MH63	osa00904	Diterpenoid biosynthesis	1.24E‐03	7
		osa00360	Phenylalanine metabolism	5.10E‐03	7
		osa00940	Phenylpropanoid biosynthesis	3.40E‐02	11
		osa00053	Ascorbate and aldarate metabolism	3.40E‐02	5
		osa04626	Plant–pathogen interaction	3.40E‐02	10
	SY63/MH63	osa04075	Plant hormone signal transduction	5.95E‐03	16
		osa04016	MAPK signaling pathway‐ plant	1.32E‐02	10
		osa00904	Diterpenoid biosynthesis	3.03E‐02	5
	JY63/MH63	osa00904	Diterpenoid biosynthesis	8.33E‐04	6
		osa04016	MAPK signaling pathway ‐ plant	1.36E‐02	8
	JH1/XS11	osa00360	Phenylalanine metabolism	2.95E‐03	5
		osa00940	Phenylpropanoid biosynthesis	1.14E‐02	7
	T202/XS11	osa03010	Ribosome	5.19E‐03	22
		osa04075	Plant hormone signal transduction	2.19E‐02	16
Group 3	CJ03/XS11	–			
	SY63/JY63	–			
	SY63/XIY63	–			
	SY63/XXY63	–			
	JY63/XIY63	–			
	JY63/XXY63	–			
	XIY63/XXY63	osa04626	Plant–pathogen interaction	1.02E‐03	8
		osa00360	Phenylalanine metabolism	1.41E‐02	4
	T202/CJ03	–			
	T202/JH1	osa00940	Phenylpropanoid biosynthesis	3.55E‐03	15
		osa00360	Phenylalanine metabolism	3.55E‐03	8
	CJ03/JH1	osa04626	Plant–pathogen interaction	1.49E‐02	11
Group 4	T1C‐19/HH1	–			
	T1C‐19/KMD1	osa03010	Ribosome	5.50E‐09	73
	T1C‐19/KMD2	osa03010	Ribosome	1.36E‐04	59
	HH1/KMD1	–			
	HH1/KMD2	–			
	KMD1/KMD2	–			
Group 5	SY63/T202	–			
	SY63/JH1	–			
	SY63/CJ03	–			
	SY63/XS11	–			
	JY63/T202	–			
	JY63/JH1	osa03010	Ribosome	8.26E‐04	47
	JY63/CJ03	–			
	JY63/XS11	osa03010	Ribosome	5.10E‐08	58
	XIY63/T202	–			
	XIY63/JH1	osa03010	Ribosome	3.56E‐19	99
	XIY63/CJ03	–			
	XIY63/XS11	osa03010	Ribosome	5.07E‐16	98
	MH63/T202	–			
	MH63/JH1	osa03010	Ribosome	3.25E‐05	59
	MH63/CJ03	–			
	MH63/XS11	osa03010	Ribosome	1.06E‐08	69
	XXY63/T202	–			
	XXY63/JH1	osa03010	Ribosome	1.67E‐05	62
	XXY63/CJ03	–			
	XXY63/XS11	osa03010	Ribosome	1.00E‐07	69

–, No significantly enriched pathways; MAPK, mitogen‐activated protein kinase.

We also performed KEGG pathway enrichment analyses of the unique and shared DEGs of comparisons showed in Figure 2(d–f). Unique DEGs in KMD2/XS11 are significantly enriched in phenylalanine, tyrosine and tryptophan biosynthesis, shared DEGs in GJ rice lines are significantly enriched in beta‐alanine metabolism, and unique DEGs in cross‐breeding are significantly enriched in plant hormone signal transduction (Table S5). Not surprisingly, these pathways are involved in plant basic metabolic processes, and none of the pathways mentioned are involved in detrimental pathways. There were no significant enriched pathways of the unique DEGs in the other subgroups (Table S5).

### Metabolomic differences in leaves among rice lines

In the present study, we profiled the metabolic changes in leaves of all 13 rice lines. In total, 821 metabolites were detected, with a range from 812 to 819 in XIlines, and from 805 to 809 in GJ lines (Table S6). The 821 metabolites were grouped into 32 classes, with the majority belonging to the classes of flavone, organic acids and flavone C‐glycosides (Figure S2 and Table S6). In addition, few metabolites from the classes of proanthocyanidins, pyridine derivatives, terpenoids and nicotinic acid derivatives were detected (Figure S2 and Table S6).

**Figure 3:**
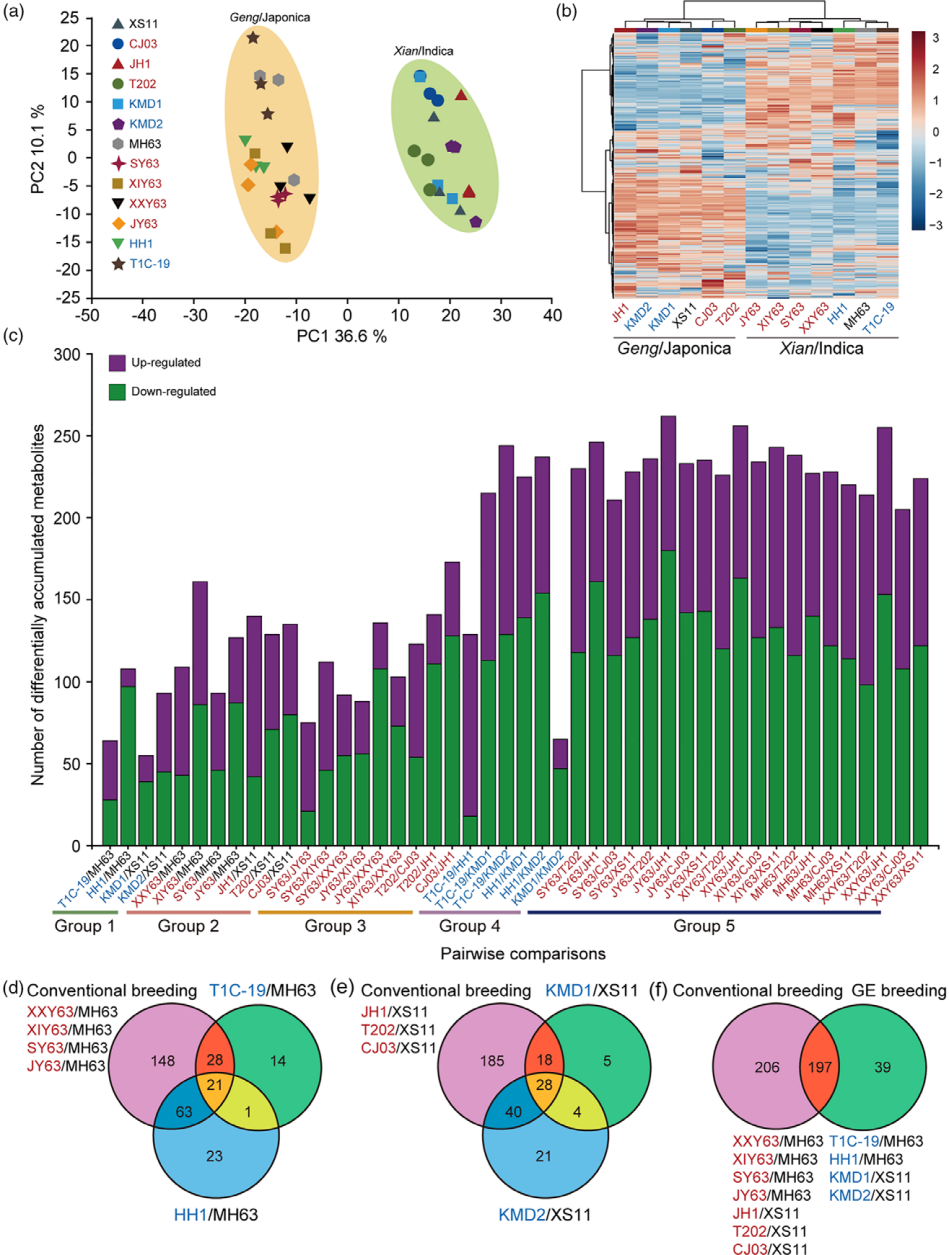
Overall description of metabolome data. (a) Principal components (PCs) analyses of metabolite accumulation levels in leaves of 13 rice lines. Score plot of the first two PCs with the explained variance. (b) Hierarchical clustering of 13 rice lines using metabolite accumulation data. In the heatmap, each rice line is visualized in a single column and each metabolite is represented by a single row. Metabolite accumulation are shown in different colors, where red indicates high abundance and low relative expression is shown in blue (color key scale right of the heat map). metabolites and samples are clustered using Euclidean distance measure and Ward clustering algorithm using Euclidean distance measure and Ward clustering algorithm. (c) Pairwise comparisons of differentially accumulated metabolites between different rice lines. (d–f) Venn diagrams depicting the unique and shared differentially accumulated metabolites among *Xian*/Indica subspecies (d), among *Geng*/Japonica subspecies (e), and between lines obtained by conventional breeding or genetic engineering (GE) breeding (f).

To investigate the total composition of the metabolomics difference in leaves of the different rice lines, a PCA plot for the accumulation of metabolites was conducted. The PCA score scatter plots for all samples are shown in Figure 3(a), where the abscissa and the ordinate represent the scores of PC1 and PC2, respectively. The first two PCs explain 36.6% and 10.1% of the total variance, respectively. The PC1 showed a clear separation between rice lines with different genetic backgrounds. For both rice subspecies (XI and GJ), the first two PCs could not separate the GE lines or conventionally bred lines from their parental lines, and GE lines from conventionally bred lines (Figure 3a). Consistently, clustering analysis of the 821 metabolites from the 13 rice lines showed that the XI and GJ subspecies were clustered into distinct groups (Figure 3b). Specifically, Bt rice lines clustered more closely with their common parental lines than the conventional cross‐breeding lines in both XI and GJ genetic background.

**Figure 4:**
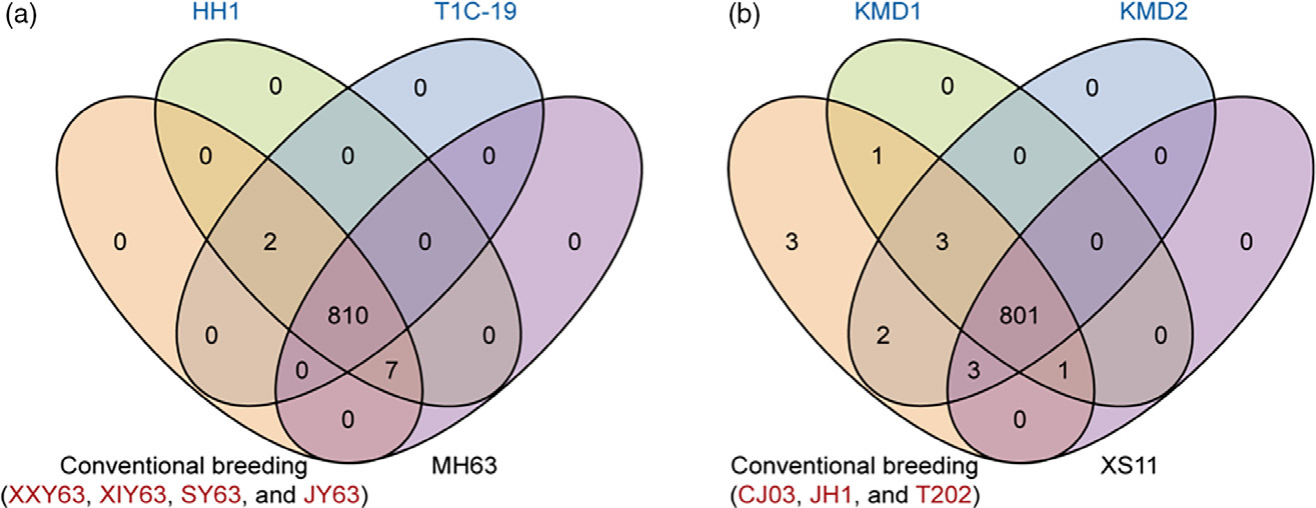
Composition analyses of metabolites detected in 13 rice lines. Venn diagrams depicting the unique and shared metabolites among *Xian*/Indica (a) and *Geng*/Japonica (b) rice subspecies.

For qualitative analysis of the metabolites, we pooled the metabolites detected in the leaves of XI or GJ rice lines that were developed by conventional cross‐breeding methods (Figure 4a,b). The Venn diagram shows that XI rice lines developed by conventional breeding or GE shared 810 metabolites (Figure 4a). Pairwise comparisons revealed that there were two new metabolites, (+)‐piperitol (hydroxycinnamoyl derivatives) and 1,4‐dihydro‐1‐methyl‐4‐oxo‐3‐pyridinecarboxamide (pyridine derivatives) detected in the GE rice lines T1C‐19 and HH1 relative to their parental line MH63 (Figure 4a and Table S7). However, the two compounds were not specific to GE rice lines and were detected in the conventionally bred lines XXY63, XIY63, SY63 and JY63 (Figure 4a and Table S7). Similarly, there were 801 common metabolites detected in GJ rice lines that were developed either by conventional breeding or GE (Figure 4b). Likewise, pairwise comparisons showed that the few new compounds, that is, 2‐methoxybenzoic acid, epigallate catechin gallate, icariin, cyanidin, procyanidin B2 and pinocembrin in GE rice lines (KMD1 and KMD2) were also detected in conventionally bred lines (JH1, T202 and XS11) (Figure 4b and Table S7). As expected, pairwise comparisons indicated that rice lines derived from XI and GJ subspecies, respectively, have more diversity in their metabolite compositions (Table S7).

To identify the differentially accumulated metabolites (DAMs) in leaves among different rice lines, we further compared the metabolite levels in the 13 lines. In total, 55–262 DAMs were identified representing a range from 7.26% to 46.87% in the total detected metabolites in each of the 44 comparisons (Figure 3c and Table S8). Pairwise comparisons showed that the percentage of DAMs from the total detected metabolites ranged from 7.26% to 15.15% in the comparisons between GE rice and their non‐GE parents, from 12.77% to 24.39% in the comparisons between conventional breeding rice lines and their parents, from 10.08% to 26.91% in the comparisons among conventional breeding rice lines with the same parents, from 8.68% to 42.29% in the comparisons among Bt rice lines, and from 30.58% to 46.87% in the comparisons among natural genotypic rice lines (Table S8). In most cases, a higher proportion of downregulated DAMs compared with upregulated DAMs was recorded (Figure 3c). In addition, the number of DAMs between GE lines and non‐GE parental rice lines were, in all cases, less than those between GE rice lines and conventionally cross‐breeding rice lines (Figure S2b).

Venn diagrams were also used for comparing the distribution of DAMs among the different rice lines (Figure 3d–f). As done for the transcriptome analyses, we pooled the DAMs of conventional cross‐breeding lines with the same parents. The results showed that a large number of unique DAMs were detected in XI conventional cross‐breeding lines compared with that in rice lines developed by GE (Figure 3d). A similar scenario was also found in GJ rice lines in which 185 unique DAMs were detected in conventional cross‐breeding lines, and in total, five and 21 unique DAMs were found in the comparisons of KMD1 and KMD2 with XS11, respectively (Figure 3e). Although conventional cross‐breeding and GE breeding shared 197 DAMs, there were still 206 unique DAMs that were identified in the former, which is over fivefold more than that in GE breeding (39) (Figure 3f).

KEGG pathway enrichment analysis showed no pathways except for the flavonoid biosynthesis and the flavone and flavonol biosynthesis were significantly enriched in the DAMs of group comparisons (Table S9). Between GE and their non‐GE counterparts, the DAMs were significantly associated with only flavonoid biosynthesis, and both the flavonoid biosynthesis pathway and the flavone and flavonol biosynthesis were found to be enriched in DAMs between conventionally bred lines and their parental lines (Table S9).

## DISCUSSION

Transcriptomics, proteomics and metabolomics have been widely used in assessing the effects of GE in crop breeding at the mRNA, protein and metabolite levels (NASEM, 2016). However, in most of these studies, analyses were restricted to comparing one GE line and its non‐GE counterpart, and the results typically revealed some degree of variation. As the exact functions of the majority of genes, proteins and metabolites in a plant cell are not clear, such results can hardly indicate the biological relevance of the detected changes, and cannot reflect whether the observed differences were caused by the GE or fall into the potential variation range caused by conventional breeding (Ricroch *et al*., 2011; Gong and Wang, 2013; Herman and Price, 2013; NASEM, 2016; Raybould and Macdonald, 2018). We thus attempted to test in rice whether GE breeding will result in novel or greater unintended effects in crops relative to conventional cross‐breeding using, in total, four GE and nine conventional rice lines. We believe that rice is a good model plant for those investigations, as it is an important food crop and has been widely used in the past for plant biology studies, including omics analyses.

Previous studies have suggested that environmental stresses brought more biological variation than transgenesis and genetic background in plants (Ouakfaoui and Miki, 2005; Batista *et al*., 2008; Montero *et al*., 2011; Asiago *et al*., 2012; Batista *et al*., 2017; Wang *et al*., 2018b; Tan *et al*., 2019). In the present study, the seeds of all 13 rice lines were prepared in the same way and were cultivated and managed under the same conditions in a greenhouse to minimize the effects of varying environmental conditions. In addition, the middle part of the top second fully expanded leaf of tillering plants from all rice lines were sampled on the same day to reduce the potential effects caused by plant developmental stages. Thus, the differences in rice leaf transcriptome and metabolome profiles should largely reflect the genotype differences of the tested rice lines. Furthermore, to increase the comparability, the conventional and GE rice varieties used in this study have the same parental line either MH63 or XS11 to ensure that they had a similar genetic background (Figure 1).

Both PCA and hierarchical cluster analyses of the raw datasets showed a distinct separation between samples with GJ and those with XI genetic background at both transcriptome and metabolome levels, irrespective of the breeding method used to develop the rice lines (GE or conventional cross‐breeding). This result is consistent with previous studies that the GJ and XI subspecies have a clear distinction in gene expression, protein characterization, metabolite accumulation and even in root microbiota composition (Jung *et al*., 2013; Hu *et al*., 2014; Wang *et al*., 2018a; Zhang *et al*., 2019). In plants, natural variation in the transcriptome and metabolome is very common (Catchpole *et al*., 2005; Batista and Oliveira, 2010; Baniasadi *et al*., 2014; Wang *et al*., 2018a,b). Studies with wheat and barley showed a clear discrimination between different conventionally bred varieties but no discrimination between GE and non‐GE counterparts at transcriptomic or metabolomic levels (Ioset *et al*., 2007; Kogel *et al*., 2010). Similar results were also found in Embrapa 5.1 common bean with a higher similarity between a GE variety and its non‐GE near‐isogenic line than between two common bean varieties in both leaf and grain proteomic profiles (Balsamo *et al*., 2015; Valentim‐Neto *et al*., 2016). The current finding together with previous results suggest that the intrinsic differences in genetic background bring much greater variation in plant transcriptome and metabolome than by the introduction of foreign genes by genetic manipulation or cross‐breeding methods.

As expected, pairwise comparisons did reveal some differences between the GE and parental non‐GE rice lines in respect to gene expression and metabolite accumulation as reported previously for GE maize and soybean (Cheng *et al*., 2008; Coll *et al*., 2008; Hao *et al*., 2017; Wang *et al*., 2018b). However, the number of DEGs observed when comparing GE and non‐GE rice lines were comparable with those present when comparing conventionally bred rice lines and their parental lines (Figures 2 and 3). An exception was the comparison between HH1 and MH63 where a higher variation in gene expression was detected than between conventionally bred rice lines and their parental lines. However, this increase in variation was not supported by DAMs, i.e., the number of DMAs between HH1 and MH63 was much lower than that in most of the comparisons between conventionally bred rice lines and their parental lines. In addition, compared with the common parental line XS11, KMD2 had nearly threefold DEGs and twofold DAMs than KMD1, although the two GE lines have a matched genotype, express the same gene and were created with the same gene transformation process (Table S10). These results thus suggest that genetic changes commonly occur during the plant breeding process whether done by GE or conventional crossing, and that the extent of those changes seems not always relevant to the extent of metabolomic change in the rice plant. Therefore, we suggest that the transcriptomics results should be integrated with results from other omics approaches such as metabolomics and proteomics to show more comprehensively the possible unintended effects caused by the plant breeding process.

The Venn diagrams of genotype‐specific DEGs and DAMs showed that both GE and the conventional breeding processes can result in a large number of DEGs and DAMs in plants. Although there were large overlapping sets of DEGs and DAMs caused by both plant breeding processes, we did find some DEGs and DAMs specifically caused by GE that were outside of the range of variation in conventional breeding rice lines (Figures 2f and 3f). It implies that the GE process may bring different stresses on the host genome relative to conventional cross‐breeding, namely two plant breeding processes may lead to the variations in gene and metabolite at different levels. However, to conclude this, a larger set of conventionally bred rice varieties would have to be analyzed. Interestingly, we detected more DAMs in plants caused by conventional breeding than the genetic breeding process. This may imply that conventional cross‐breeding required multiple repeated crosses between two or more breeding lines, thus causing more changes on both genotypic and phenotypic levels (Coll *et al*., 2009).

Our GO enrichment analyses indicated that the DEGs in different comparisons were involved in “DNA integration.” It can be speculated that the differences in gene expression brought by either GE or conventional breeding, or due to natural variation are all associated with changes in DNA sequence. There were many DEGs and DAMs detected not only between GE rice lines and their non‐GE counterparts, but also between conventionally bred rice lines and their parental lines (Figure 2f and 3f). However, the DEGs detected between the GE rice lines and their non‐GE counterparts were significantly enriched only in photosynthesis, but the DEGs between conventionally bred rice lines and their parental lines were significantly enriched within multiple pathways. Likewise, the DAMs were only found to be enriched in the flavonoid biosynthesis pathway between the GE rice lines and their non‐GE counterparts, but the DAMs between conventionally bred rice lines and their parental lines were enriched in both the flavonoid biosynthesis pathway and the flavone and flavonol biosynthesis pathway. These results may suggest that GE does not bring unique effects on plant pathways compared with conventional cross‐breeding. All four GE rice lines used in the current study expressed foreign *Bt cry* genes conferring resistance to insects. The expressed crystal proteins are not native to plants and exert no known metabolic activity in rice plants (Wang *et al*., 2018b; Fu *et al*., 2019). While if the inserted genes in GE plants are involved in plant metabolic pathways, the results may be different (Wang *et al*., 2018b, 2019). For example, there is a case in GE plants that are tolerant to the herbicide glyphosate, as the tolerance is conferred by introducing a glyphosate‐insensitive version of the target enzyme 5‐enolpyruvoylshikimate‐3‐phosphate synthase, which is a key enzyme in the shikimate pathway.

The substantial equivalence concept is an important part in the safety assessment of GE crops (Ricroch *et al*., 2011; Asiago *et al*., 2012). Our metabolomics analysis did detect some compounds, including (+)‐piperitol, 2‐methoxybenzoic acid, epigallate catechin gallate, icariin, cyanidin, procyanidin B2 and pinocembrin in GE rice tissues that had not been detected in the non‐GE parental plants. However, all of these compounds were also found in several of the conventionally bred rice lines. As the conventionally bred rice lines have been widely planted and have a long history of safe use, these compounds will not bring detrimental effects on human health and the environment. Our results further strengthen the fact that assessment of unintended effects of GE plants cannot simply rely on the comparison between GE plants and their parental lines but should include a set of conventionally bred cultivars that represent the range of genetic and phenotypic diversity in the crop (NASEM, 2016). The integrated application of multi‐omics approaches can more comprehensively reflect changes in the plants and their biological relevance.

In conclusion, we successfully employed RNA‐seq and HPLC‐MS technology to investigate the changes in gene expression and metabolite accumulation in 13 rice lines developed by conventional cross‐breeding or GE. Our results demonstrate that the emerging ‐omics approaches can provide a valid way for identifying unintended effects of GE varieties. The current findings suggest that both breeding methods can result in potential changes at transcriptomic and metabolic levels, and it appears that GE does not cause unintended effects that go beyond conventional cross‐breeding. Although we did detect DEGs and DAMs specifically caused by GE that were outside of the range of variation in conventional breeding rice lines, this could be due to the limited number of conventionally bred rice lines involved in the study and it can be expected that the natural variation in DEGs and DAMs is actually much larger. Therefore, a comprehensive range of variation at transcriptome, metabolome and proteome levels in commercially conventionally bred cultivars of a crop species would have to be established before those analyses are deployed for identifying the unintended effects in GE varieties.

## EXPERIMENTAL PROCEDURES

### Plant materials

In total, 13 rice lines (*Oryza sativa*) including four lines developed by GE expressing *Bt* genes and nine lines obtained by conventional cross‐breeding were used in this study (Figure 1 and Table S10). The Bt rice lines include T1C‐19, which expresses a synthesized *cry1C* gene driven by the maize *ubiquitin* promoter; the Bt rice line HH1 expresses a fused *cry1Ab/Ac* gene driven by the rice *actin1* promoter. Both T1C‐19 and HH1 share the same non‐transformed near isoline MH63, which is an elite XI restorer line for cytoplasmic male sterility in China (Wu *et al*., 2011). The other two Bt rice lines are KMD1 and KMD2, and their corresponding non‐transformed near isoline XS11. XS11 is a GJ rice line widely cultivated in China. KMD1 and KMD2 are independent homozygous events containing the same synthetic *cry1Ab* gene under the control of the maize *ubiquitin* promoter. All the Bt rice lines were developed by *Agrobacterium tumefaciens* infection. Laboratory and field experiments indicated that all four Bt rice lines are highly resistant to target lepidopteran insects (Liu *et al*., 2016). Among the nine non‐Bt rice lines used in this study, four are XI lines (XY63, JY63, XXY63 and SY63) obtained by crossing MH63 with different rice varieties, and three are GJ lines (CJ03, JH1 and T202) obtained by crossing XS11 with other rice varieties (Figure 1). The seven cross‐pollination rice lines are all approved by provincial or national registration committees for crop varieties in China. Among these lines, SY63 was widely planted with the highest total area of 62.88 million hectares from 1984 to 2009 (Wu *et al*., 2011).

### Plant growth condition and tissue sampling

Rice seeds were dehusked, surface‐sterilized using 75% ethanol for 5 min and washed with sterilized water. The seeds were then soaked in 4% sodium hypochlorite solution for 30 min and washed again with sterilized water. Subsequently the surface‐sterilized rice seeds were germinated on half‐strength Murashige and Skoog medium in a climate chamber at conditions of 28 ± 1°C, a 16‐h light/8‐h dark photoperiod and 75% ± 5% relative humidity. A week later, seedlings were transplanted into individual plastic pots (8 cm × 10 cm, diameter × height) containing a mixture of peat and vermiculite in a 3:1 ratio (Meihekou Factory, Meihekou, China). All potted plants were placed in a cement pool that was maintained in a glasshouse at the Langfang Experimental Station of the Institute of Plant Protection, Chinese Academy of Agricultural Sciences (CAAS). The growth conditions were set as 28 ± 2°C, 65% ± 10% relative humidity and a 16‐h light/ 8‐h dark photoperiod. Nitrogenous fertilizer Sakefu (N [20%], P_2_O_5_ [20%], K_2_O [20%]) and other microelements (Sino‐Arab Chemical Fertilizer Co., Ltd, Qinhuangdao, China) was applied once a week. Five weeks later, during the rice plants tillering stage, leaf samples were collected for the analyses. From each plant, a leaf section (approximately 2 cm) was sampled from the middle part of second leaf blade from top. Samples from five plants were pooled together as one biological replicate, and three replicates were collected for each rice line. The leaf samples were immediately frozen in liquid nitrogen and stored at −80°C for further extraction and analyses.

### RNA extraction, library preparation and RNA‐sequencing

Total RNA was isolated using the TRIzol reagent (Invitrogen, Carlsbad, CA, USA) and treated with RNase‐free DNase I (NEB, Ipswich, MA, USA) to remove any genomic DNA. In total, 3 μg of total RNA per sample was used for the preparation of RNA‐seq library. Sequencing libraries were prepared using a NEBNext^®^ Ultra™ RNA Library Prep Kit for Illumina^®^ (NEB) and were sequenced on the Illumina Hiseq 4000 platform (Illumina, San Diego, CA, USA) according to the manufacturer's instructions by Nonogene (Beijing, China).

For transcriptome data, raw Illumina data of fastq format were first processed using in‐house perl scripts. After removing reads containing adaptors, reads containing poly‐N and low‐quality reads from raw data, we obtained the clean data. The Q20, Q30, guanine‐cytosine content and sequence duplication level of the clean data were calculated. The clean reads were aligned to the reference genome IRGSP‐1.0 (https://rapdb.dna.affrc.go.jp) using HISAT2 tools (version 2.09) (Kim *et al*., 2015). feature counts (v1.5.0‐p3) (Liao *et al*., 2014) was used to count the read numbers mapped to each gene. Gene expression levels were estimated using fragments per kilobase of transcript per million mapped reads based on the length of the gene and reads count mapped to this gene. Differentially expressed genes analysis was performed using the deseq2 R package (Love *et al*., 2014). The resulting *P* values were adjusted using the Benjamini and Hochberg’s approach for the control of the false‐discovery rate. Genes with fold‐change ≥2 or ≤0.5 and an adjusted *P* < 0.05 were considered as DEGs. GO and KEGG pathway enrichment analysis were performed using the clusterprofiler R package with an false‐discovery rate adjusted *P* < 0.05 (hypergeometric test) as a cutoff (Yu *et al*., 2012).

### Metabolite profiling

#### Metabolite extraction process

Freeze‐dried leaf samples of the 13 rice lines were grinded using a mixer mill (MM 400; Retsch, Haan, Germany) with a zirconia bead for 1.5 min at 30 Hz. For each sample, 100 mg of leaf power was weighted and mixed with 1 ml of 70 % aqueous methanol for extraction overnight at 4°C. Following centrifugation at 10 000 ***g*** for 10 min the extracts were absorbed (CNWBOND Carbon‐GCB SPE Cartridge, 250 mg, 3 ml; ANPEL, Shanghai, China) and filtrated with a membrane (SCAA‐104, 0.22 μm pore size; ANPEL, Shanghai, China), and subsequently stored in a glass vial before analysis.

#### HPLC and ESI‐Q TRAP‐MS/MS running conditions

Metabolite identification and quantification were performed using an LC‐electrospray ionization‐MS/MS (LC‐ESI‐MS/MS) system: HPLC, Shim‐pack UFLC SHIMADZU CBM20A system, Kyoto, Japan; MS, Applied Biosystems 4000 Q TRAP, Foster City, CA, USA) as previously described (Chen *et al*., 2013). The HPLC analytical conditions were set as follows: the chromatographic column was a Waters ACQUITY UPLC HSS T3 C18 column (1.8 μm, 2.1 mm × 100 mm). The solvent system included mobile phase A, 0.04% acetic acid in water, and mobile phase B, 0.04 % acetic acid in acetonitrile. The gradient program was set at 95:5 V(A)/V(B) at 0 min, 5:95 V(A)/V(B) at 11 min, 5:95 V(A)/V(B) at 12 min, 95:5 V(A)/V(B) at 12.1 min, 95:5 V(A)/V(B) at 15 min. The temperature of the column was 40°C, the flow rate was 0.4 ml min^−1^, and the injection volume was 5 μl. The effluent was then connected to an ESI‐triple quadrupole‐linear ion trap (Q TRAP)‐MS.

Linear ion trap and triple quadrupole (QQQ) scans were acquired on a Q TRAP‐MS, API 4500 Q TRAP LC/MS/MS System, equipped with an ESI Turbo Ion‐Spray interface, operating in a positive ion mode and controlled by the Analyst 1.6 software (AB Sciex, Darmstadt, Germany). The ESI source operation was set with the following parameters: ion source, turbo spray; source temperature, 550°C; ion spray voltage, 5.5 kV; ion source gas I, gas II, and curtain gas were set at 55, 60 and 25 psi, respectively; the collisionally activated dissociation gas was high. Instrument tuning and mass calibration were performed with 10 and 100 μm polypropylene glycol solutions in QQQ and linear ion trap modes, respectively. The metabolites were identified according to the secondary spectrum information. QQQ scans were acquired as multiple reaction monitoring mode experiments with the collision gas (nitrogen) set to 5 psi.

#### Acquiring metabolic data

For metabolome data, metabolite identification was performed according to MWDB (metware database; MetWare, Wuhan, China) and publicly available metabolite databases including MassBank (http://www.massbank.jp/), KNAPSAcK (http://kanaya.naist.jp/KNApSAcK/), HMDB (http://www.hmdb.ca/), MoTo DB (http://www.ab.wur.nl/moto/) and METLIN (http://metlin.scripps.edu/index.php). The quantification of metabolites was carried out using a scheduled multiple reaction monitoring method (Chen *et al*., 2013).

### Data analysis

Raw data associated with gene expression and metabolites accumulation were median‐normalized, log‐transformed and auto‐scaled using metaboanalyst 4.0 (Chong *et al*., 2018). Hierarchical clustering heat map was created using metaboanalyst 4.0 with Euclidean distance measure and Ward clustering algorithm. The normalized data were fed to simca 14.1 software (Umetrics, Umea, Sweden) for PCA. Orthogonal projections to latent structures discriminant analysis was conducted for identifying DAMs between any two rice lines using simca 14.1 software. The variable importance in the projection values ≥1.0 generated in orthogonal projections to latent structures discriminant analysis processing was first used as a criterion for the selection of DAMs. The fold‐changes ≥2 or ≤0.5 were defined as DAMs. Fisher's exact test was performed to identify the significant KEGG pathways related to the DAMs with an adjusted *P* < 0.05.

## CONFLICT OF INTEREST

The authors declare that they have no competing interests.

## AUTHOR CONTRIBUTIONS

YL conceived the idea. YL, QL and JR designed the study. QS performed the experiments. QL, YL and VT analyzed the data. QL, YL, XY, VT, YP and JR wrote the manuscript. All authors have read and approved the manuscript for publication.

### OPEN RESEARCH BADGES

This article has earned an Open Data badge for making publicly available the digitally‐shareable data necessary to reproduce the reported results. The transcriptome sequencing data can be accessed on the NCBI's Gene Expression Omnibus (GEO) (GSE152572). Other relevant data can be found with the article and its supporting materials.

## Supporting information


**Figure S1.** Number of differentially expressed genes (a) and differentially accumulated metabolites (b) in pairwise comparisons of Bt rice lines and conventional cross‐breeding non‐Bt rice lines.

**Figure S2.** Classification of the 821 detected metabolites in the leaves of 13 rice lines into major classes.

**Table S1.** Summary of RNA‐seq and transcriptome mapping results.
**Table S2.** Expression of detected genes in different rice lines.
**Table S3.** Number of DEGs and percentage of the DEGs on the total detected genes in pairwise comparisons of different rice lines.
**Table S4.** Gene Ontology enrichment analyses of DEGs among different comparisons.
**Table S5.** Kyoto Encyclopedia of Gene and Genomes pathway enrichment analyses of putative unique and common DEGs in comparisons among different rice lines.
**Table S6.** Metabolic profiles for compounds accumulated in different rice lines.
**Table S7.** Pairwise compositional comparison of compounds detected in different rice lines.
**Table S8.** Percentage of differentially accumulated metabolites on the total detected metabolites in pairwise comparisons of different rice lines.
**Table S9.** KEGG pathway enrichment analysis of significantly differentially accumulated metabolites.
**Table S10.** Rice lines used in this study.

## Data Availability

The transcriptome sequencing data used in this study were submitted to the NCBI's Gene Expression Omnibus (GEO) under accession number GSE152572. Other relevant data can be found within the manuscript and its supporting materials.
