# Supplementary figures and images for: Plant breeding involving genetic engineering does not result in unacceptable unintended effects in rice relative to conventional cross‐breeding
Source: Plant J. 2020 Jul 19;103(6):2236–49. doi: 10.1111/tpj.14895 (PMC7540705; doi:10.1111/tpj.14895)

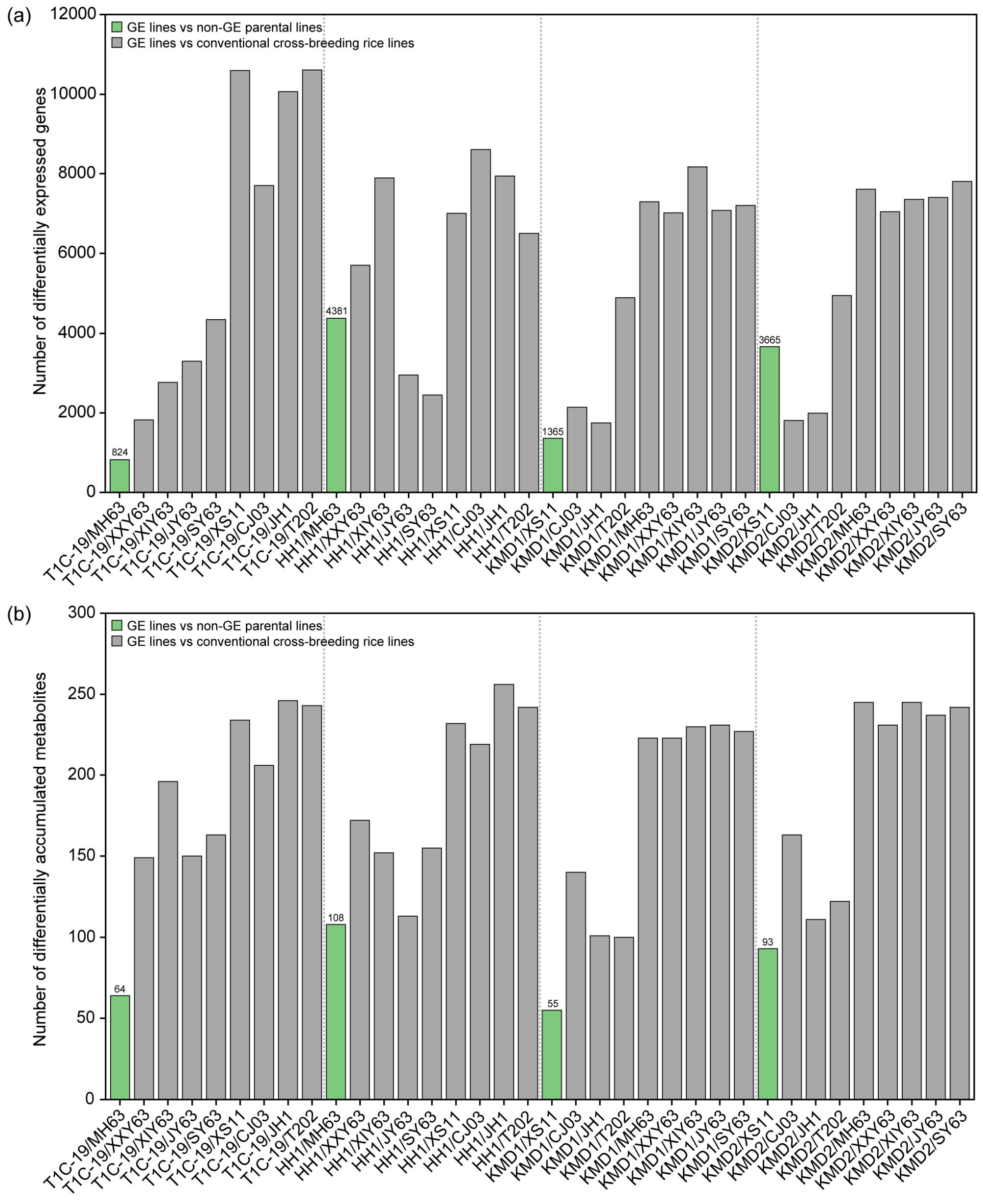

Supplement: Supplementary file 1 — Figure S1. Number of differentially expressed genes (a) and differentially accumulated metabolites (b) in pairwise comparisons of Bt rice lines and conventional cross‐breeding non‐Bt rice lines. [file TPJ-103-2236-s001.tif]

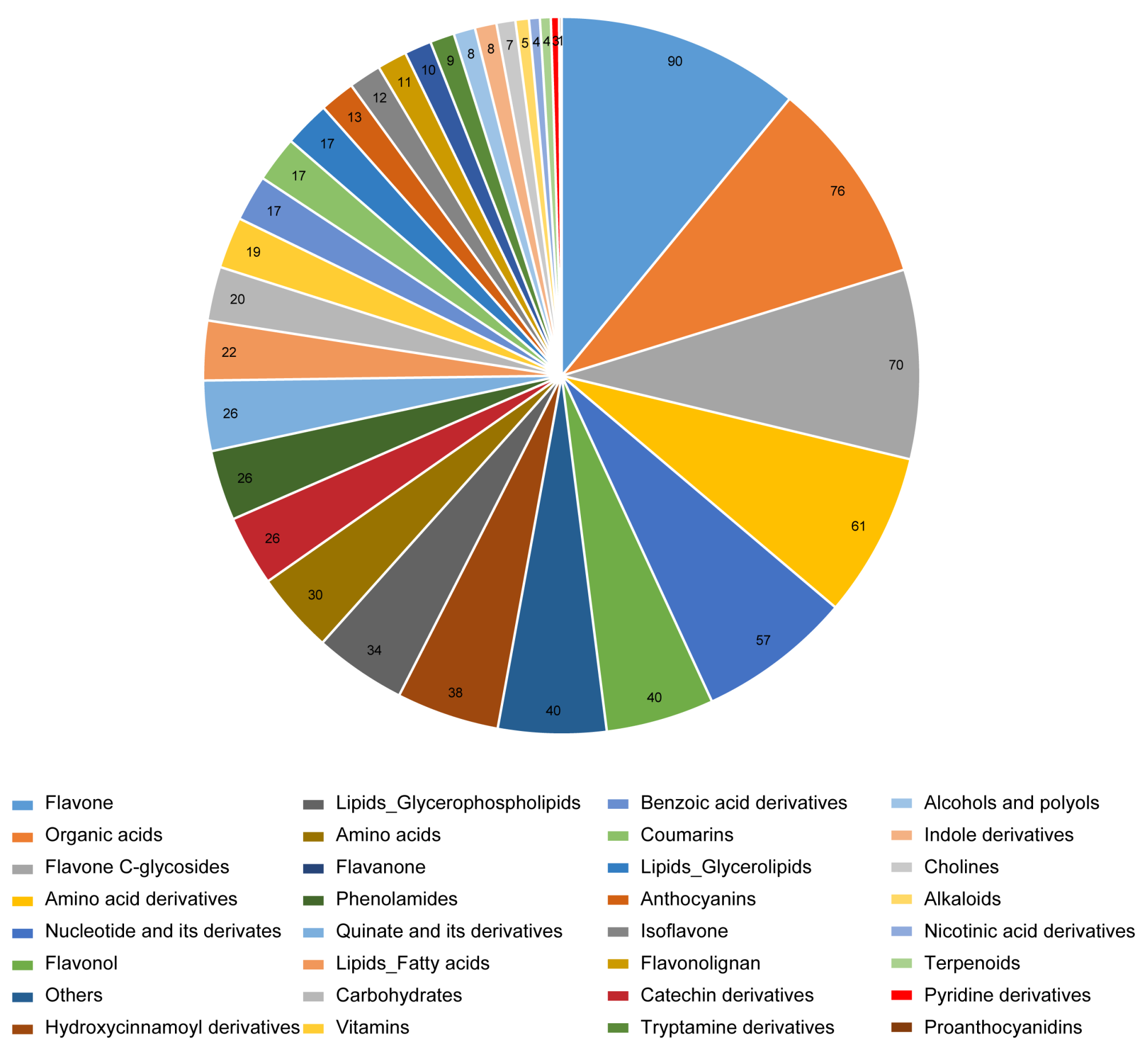

Supplement: Supplementary file 2 — Figure S2. Classification of the 821 detected metabolites in the leaves of 13 rice lines into major classes. [file TPJ-103-2236-s002.tif]
